# “I think to myself ‘why now?’” – a qualitative study about endometriosis and pain in Austria

**DOI:** 10.1186/s12905-023-02576-w

**Published:** 2023-08-04

**Authors:** Manuela Gstoettner, René Wenzl, Ines Radler, Margret Jaeger

**Affiliations:** 1 Department of Obstetrics and Gynecology, Medical University of Vienna, Waehringer Gürtel 18-20, Vienna, 1090 Austria; 2 Krankenhaus der Barmherzigen Schwestern, Seilerstätte 4, Linz, 4010 Austria; 3 Research Department of Education Centre of Social Fund Vienna, Schlachthausgasse 37, Vienna, 1030 Austria

**Keywords:** Endometriosis, Pain, Qualitative methods, Social environment, Austria

## Abstract

**Background:**

Endometriosis is a chronic, benign, and oestrogen-dependent condition and about 10–15% of all women of reproductive age are affected by endometriosis worldwide. It is not curable and pain is one of the most common symptoms of endometriosis and leads to low quality of life in affected women. To our knowledge, in German-speaking countries, no studies with qualitative methods approaches are available concerning women who suffer from pain caused by endometriosis and possible associated coping strategies. Our study aims to familiarise ourselves with the individual pain experience of selected women who suffer from endometriosis in Austria and their coping strategies.

**Methods:**

A qualitative study design was based on problem-centred interviews for data collection and qualitative content analysis for data analysis. The research participants were women aged between 18 and 55 diagnosed with endometriosis and living in Austria. The interview period was from 27 February to 26 March 2019 and interviews lasted between 50 and 75 min.

**Results:**

Eight categories were formulated, of which category 3 (thoughts and feelings regarding endometriosis and pain - ‘why?**’**), category 5 (effects and changes caused by endometriosis and pain – ‘quality of life’), category 7 (taboos – ‘don`t talk about it’), and category 8 (talking about it – ‘contact with others in the same position’) were relevant for this article. The remaining four categories [1–4] have already been published elsewhere.

**Conclusion:**

Our data show that the social environment plays a fundamental role in coping strategies concerning pain caused by endometriosis. Women in our study reported that exchange with peers offers support. This opens a door for information events, patient organizations like support groups, and the inclusion of these in the supporting system. Involving occupational medicine and workplace health promotion departments in companies should be further goals to support affected women.

## Background

Endometriosis, a chronic, benign, and oestrogen-dependent condition, is a disease, where endometrium grows outside the uterine cavity [1]. Worldwide about 10–15% of all women of reproductive age are affected by endometriosis – in absolute numbers, this means around 176 million women worldwide [2, 3, 5].

Unfortunately, until today, the cure is not attainable [1]. Pain is one of the most common symptoms of endometriosis and leads to low quality of life in affected women [6].

A literature review showed some qualitative studies dealing with pain experienced by women suffering from endometriosis [4, 7–10]. Most of the affected women have problems in private, social, or occupational live caused by symptoms due to this disease [2, 4, 6, 7, 11–14].

To our knowledge, in German-speaking countries, no studies with qualitative methods approaches are available concerning women who suffer from pain caused by endometriosis and possible associated coping strategies. The aim of our study is therefore – based on interviews and qualitative content analysis [15]– to familiarise ourselves with the individual pain experience of selected women who suffer from endometriosis in Austria and their coping strategies.

Different theories exist about the causes of endometriosis. The implantation theory relates to menstrual tissue from the endometrium carried backwards through the fallopian tubes. This tissue implants into the peritoneum or in the organs of the lower pelvis. However, this phenomenon is physiological in nine out of ten women and does not completely explain Sampson’s theory elaborated in the 1920s. On the other hand, the metaplasia theory states the development of endometriotic cells that arise in locations outside the uterus [16].

Furthermore, it is discussed whether uterine tissue can be transported via lymph vessels [17]. Another possible relevant aspect could be an altered immune system. The malfunctions of cellular immunity in the peritoneum insufficient immune monitoring and could be a causal effect [18]. Three types of endometriosis have been classified: peritoneal endometriosis, endometriotic cysts, and deep-infiltrating endometriosis, where the disease establishes itself in the bladder, the urethra and/or in the intestine [19].

Persons who are diagnosed with endometriosis can be symptom-free or suffer from severe dysmenorrhea, dyschezia, dyspareunia, dysuria, chronic pelvic pain and/or sterility. All of these factors can reduce the quality of life, due to pain, but also due to infertility [20]. The International Association for the Study of Pain defines pain as “*An unpleasant sensory and emotional experience associated with, or resembling that associated with, actual or potential tissue damage*” [21] and it can be divided into acute and chronic [22, 23]. Especially the chronic form can be more limiting, distressing and overwhelming than the acute pain [24]. In particular, regarding the chronic character of endometriosis, these s pain symptoms can be difficult to handle [25] as they often affect the whole lower abdomen followed by fearful expectations caused by pain. This fear leads to tension and then pain again [26].

In addition, headache, pain around the ovulation and cyclic leg pain are often accompanying symptoms [27]. These symptoms can reduce the quality of life and influence physical comfort as well as social life [6, 7, 9].

An additional factor is the delay until diagnosis which prolongs the suffering. A study, conducted in ten countries, showed a time frame from four up to ten years between first symptoms and diagnosis [28]. In the US and Great Britain, an average of 11.7 years [29] and 6.7 years are reported in Norway [30]. Germany and Austria lie between those numbers. An average of 10.4 years was found as a delay till diagnosis [31]. Another critical factor is the lack of widespread knowledge regarding endometriosis.

This disease is often not known by women who suffered from typical symptoms before they got their diagnosis. Sometimes even physicians do not take endometriosis into account if women present with lower abdominal pain [32].

## Methods

The qualitative study design was based on problem-centred interviews [33] for data collection and qualitative content analysis [15] for data analysis. This choice enabled “*the examination of subjective realities and subjective constructions of meaning and everyday theories, the description of life worlds from the inside, and the analysis of individual perspectives and opinions or motives. All of this with the goal of not only describing in detail but also to be able to understand.”* (34:2).

An interview guide was used to collect the data [34]. It was based on existing theories and followed the McGill Illness Narrative Interview (MINI) of Groleau, Young and Kirmayer (2006). Using one section of MINI (originally in English, translated to German for the study) enabled us to understand personal experiences of illness and its embedding in a cultural context and social processes. A quality assurance process was created to translate the section of questions: the research team translated the questions into German and native speakers of both languages edited them. The ten-steps-guide from Wild was used to check the quality of our translation. Besides the linguistic correctness, it was important to also draw attention to the cultural adaptation to the local situation [35].

## Material

The research participants were women aged between 18 and 55 diagnosed with endometriosis and living in Austria. The recruitment process included a call for interview partners through the Endometriosis Association Austria (EVA) and was published on their Facebook page. In addition, Facebook was also used to promote the study. Additionally, the method of snowball sampling was applied that addresses relationships with potential research partners [36].

After a first contact via phone call, ten women aged between 22 and 51 agreed to participate. The interview period was from 27 February to 26 March 2019 and interviews lasted between 50 and 75 min. Items of socio-demographic data were surveyed at the beginning (Table 1). Standard orthography rules were used for transcription [37], followed by qualitative content analysis and the building of categories, deductively and inductively [15].

Following the Data Protective Directive of the European Union [38], the transcripts will be stored on a secure server at Sigmund Freud University Linz for ten years. After transcription, the audio files were deleted [38].

All participants gave their oral and written informed consent before taking part in the study. After content analyses the individual data are presented anonymously. All women were given an individual code (P1, P2, …). The women in our study gave their consent as affected by the disease and not as patients – the interviews did not take place in healthcare institutions, so, the province’s legal provisions province and university regulations did not require approval by an Ethics Committee. All women got the advice, if needed, to contact their physicians or supporting associations like the Endometriosis Association Austria (EVA) [39].

The data analysis was carried out following the rules and techniques of qualitative content analysis and used category-led text analysis as the main tool [15]. Categories were developed deductively followed by an inductive round to assure that new topics were included. The techniques of summarising as well as explanation were used to interpret the results (ibid.).

**Table 1:** socio-demographic data of the interviewees

Participant	Occupation	Current age	Age at diagnosis (years)	Relationship status	Desire to have (more) children	Children (number)
P1	Midwife	31	around 20	married	no	2
P2	Head Secretary	25	23	relationship	yes	1
P3	management in social sector	44	around 30	married	no	2
P4	Student with part-time job	24	23	no	yes, in the future	no
P5	Fashion advisor	41	30	married	no longer	no
P6	Engineer	22	19	relationship	yes, in the future	no
P7	Student with part-time job	37	30	relationship	no	no
P8	Psychiatric nurse	28	24	married	no	2
P9	Student with part-time job	25	22	no	yes, in the future	no
P10	Radiologist	51	35	married	no	2

Women were asked about their desire to get pregnant, however, no further evaluation of this topic was a focus of our study.

## Results

The results present the feelings, experiences, and perceptions of ten Austrian women with an average age of 32.8 years. The patient journey from the first symptoms until diagnosis ranged between 2.5 months and over ten years while their average age at diagnosis was 25.6 years (Table 1).

With the aid of qualitative content analysis, eight categories including sub-categories were formulated, of which category 3 (thoughts and feelings regarding endometriosis and pain - ‘why?**’**), category 5 (effects and changes caused by endometriosis and pain – ‘quality of life’), category 7 (taboos – ‘don`t talk about it’) and category 8 (talking about it – ‘contact with others in the same position’) were relevant for this article (Table 2). The remaining four categories [1–4] have already been published elsewhere [40].

**Table 2:** Categories

Category 1: Endometriosis – ‘a little monster’
Category 2: Pain – ‘detrimental to life’
**Category 3: Thoughts and feelings regarding endometriosis and pain .- ‘Why?’**
Category 4: Dealing with endometriosis and pain – `Stay calm’
**Category 5: Effects and changes caused by endometriosis and pain – ‘Quality of life’**
Category 6: Wishes and expectations of the women – ‘Communication’
**Category 7: Taboos – ‘Don`t talk about it’**
**Category 8: Talking about it – ‘Contact with others in the same situation’**

### Category three: thoughts and feelings regarding endometriosis and pain **–** ‘Why?’

The following content covers the women’s descriptions of their thoughts and feelings regarding the disease and pain.

The participants had negative thoughts about their menstruation. This fact was illustrated, among other things, by the statement:

“*Because that was very negative for me - completely. I’ve always thought to myself, “Oh Marianna, now I’m getting her [menstrual bleeding] again.“* (P3, 44 yrs., p. 36).

Feeling intense pain caused one participant to think,

“*Please just let me die. Redeem me.*” (P7, 37 yrs., p. 81).

Thoughts also aroused regarding the inconvenience of the menstrual period occurring at a specific time.

*“Yes, I think to myself - you can really feel it, when it starts - then I just think to myself: “Please don’t. Why now?” Because sometimes it’s always when you’ve planned something or when someone comes to visit. Then I had it very often and the only thing I thought to myself was: “Please, not now.“* (P2, 25yrs., p. 18).

In addition, two affected persons reported that they were no longer able to think properly when severe pain was felt. The interviews revealed that anxiety, fear, concern, shame, sadness, anger and resentment, hatred, insecurity, helplessness and despair, nervousness, and disappointment are mentioned concerning the pain and the disease endometriosis. It was crucial to emphasize any fear of a diagnosis. Some women reported delaying a definitive examination because of uncertainty about the possible outcome.

Women feared that pain could occur anytime and anywhere with the onset of menstruation and not being able to do anything. Participants felt helpless, desperate, and insecure. In addition, uncertainty, and concern about whether activities could be carried out adequately at work despite the pain play a role. The feeling of concern that the disease will come back, for example after laparoscopic removal of endometriosis lesions, was a feeling that has been pronounced.

*“[…] it’s already in the back of my mind with “It [endometriosis] could come back.“”* (P9, 25 yrs., p. 115).

### Category five: Effects and changes caused by endometriosis and pain – ‘Quality of life`

This category summarises the experiences of the effects and changes caused by the disease.

Endometriosis and pain not only had an impact on physical concerns but also the well-being and psyche of those affected. Participants reported emotional distress, lack of motivation, fear, worries, discomfort, irritability, and anger.

*“Yes, as I said - you’re more like, yes I don’t know, a mixture between lethargic, angry and no idea. So you just don’t feel good. But you don’t feel womanly in any way either. You’re such a lump that’s really all pain right now.“* (P7, 27 yrs., p. 84).

Women reported positive developments and having learned to deal with endometriosis over time and after diagnosis, although the way was not easy. Furthermore, participants wanted to get more understanding from others in the course of the disease. A healthier and more conscious lifestyle was characterized, for example, by women enjoying the days when they are pain-free and some even doing more sports. After endometriosis and pain had often weakened women for years, some women now felt stronger, more relaxed, fitter, and healthier than when the disease began. Four women stated that they now considered themselves important, allowed themselves a break more quickly and paid better attention to themselves, their bodies, and their own needs. Gratitude for having children despite the disease was emphasized by some women. In addition, two interviewees changed their view of the desire to have children and family life. After the diagnosis, the frequency of taking painkillers was reduced as well as increased in some women. Another woman reported the fact that she changed her whole life (work, partnership, place of residence) due to endometriosis and realised, after diagnosis, who supports her and who doesn’t. The loss of the workplace and the severing of relationships were negative changes due to endometriosis and pain.

Eight out of ten women were in a heterosexual partnership or marriage at the time the data were collected. The pain during sexual intercourse often harmed the sex life. Communication with the partner turned out to be essential.

*“Sex life was often restricted. […] And it just took an insanely long time until we found out “How does it not hurt?”, “What can you do to make it not so bad?” But I think if you have a partner who loves you and who is also open, you can get used to it very easily.“* (P2, 25 yrs., p. 51).

*“The real stress was really during the sexual intercourse with my boyfriend. Because then it was really the case that we sometimes said, “Ok, I can’t do it anymore.“ Then I didn’t want to anymore either*.“ (P9, 25 yrs., p. 110).

Relationships with partners changed, positive. The positive change was mainly because the partners showed themselves to be understanding and compassionate, took their partners seriously, supported them in all matters, found encouraging words and made their partners feel that they can rely on them. However, some partners were not that understanding. One relationship even broke up because of the illness and the resulting stresses:

*“Yes, well, my relationship, which I had for seven years at the time, actually broke up because of this [due to pain], you can say. Because I’ve always been in pain and it just wasn’t accepted, because there was no reason for it now.“* (P2, 25 yrs., p. 15).

A diagnosis could be a chance for a new relationship. Three affected women were able to make an understandable explanation for their symptoms.

Women often felt restricted in the social sphere and their leisure activities, especially during menstruation. Activities were planned to take the cycle into account, which again restricted the social life and leisure time of those affected. Also, everyday activities often could not be carried out Some women only had the option of staying at home during menstrual bleeding.

*“Because it’s just really strong and because my body is basically in the same shape as a fifty or sixty-year-old - that’s how I always compare it. My body is not like that of a twenty-five-year-old, but extremely weak.”* (P2, 25 yrs., p. 19).

The effects of endometriosis and pain were also evident in eight women’s professional life. The consequences were, for example, insecurity, inhibition, anxious thoughts, or a lack of concentration in everyday work. Frequent sick leave days due to endometriosis-related pain could trigger discussions or disagreements with the employer or fear of being fired.

*“Then, of course, the fear of how that will express itself or how far one can go - sometimes it went so far that I thought, ‘Okay, I’m on the night shift at this time, that’s my period. What do I do when I get this pain during the night?”* (P1, 31 yrs., p. 6).

*“[…] and he [boss] just asked if I couldn´t do it [laparoscopy] later and I said “No, because there’s really something going on for me and I’m also in insane pain and I can’t stand it any longer.“ And then he actually fired me while I was on sick leave.“* (P2, 25 yrs., p. 24).

### Category seven: taboos – ‘Don`t talk about it’

In the following section, different taboos concerning endometriosis and associated pain are explained. Regarding the menstrual cycle, endometriosis and severe pain as taboo topics, the study participants mostly expressed diverse opinions, but different theories. For them personally, none of the three topics was taboo at the time of the interview. Men in general and especially male employers often showed less understanding of these issues, which is why communicating with them often turns out to be difficult.

However, the interviews revealed that the monthly menstrual period in adolescence or puberty was often an embarrassing topic that people felt ashamed of and therefore did not express during their adolescence. An affected woman also reported on her experience that the topic of the female cycle and the monthly menstrual period is a taboo subject, as she had never seen women talk about it among themselves, even if they were family members or close friends. Some could always talk about it with their mothers. This was not always the case, especially for those interviewees over the age of 30, which indicated a generational difference in talking about their bodies. In addition, for younger participants, the monthly menstrual was not as taboo as it used to be. Nevertheless, women had experienced that the topics tend to be taboo in many places, especially among men.

*“But then I’m there - I’m very provocative sometimes and then I talk about it on purpose because I also want them to know what we’re sometimes experiencing [during menstruation].“* (P2, 25 yrs., p. 26).

Above all, male employers showed a lack of understanding, which resulted in difficult communication with them.

“*Well, I would never say to my boss, ‘Well I - on that day today - I’m on my period right now, I’m feeling so bad, I can’t be at this meeting right now.‘ You would never say that. Well, I would have never heard of it. So that’s definitely a big taboo, really big.“* (P3, 44 yrs., p. 39–40).

One participant spoke about her experiences as a Muslim with a migration biography. She stated that she adapted culturally when communicating about menstruation.

*“For us, I think it’s also family and cultural - my parents are from Iran - it’s quite a taboo subject, it’s just menstruation and the like - just don’t talk about it.“* (P1, 31 yrs., p. 2).

Another interviewee put forward her theory that severe pain was a taboo subject because others don’t know how to deal with it.

*“I can imagine. But I think that’s more because other people can’t deal with it at all. How do you comfort someone who is in severe pain?”* (P1, 31 yrs., p. 12).

Further opinion on this is the following: Whether severe pain was a taboo subject or not depends on what was causing the pain. For example, there was more understanding of migraine or headaches than of pain due to endometriosis.

*“It has been my experience that people listen to you at the beginning and then after a week they say: ‘Yes, but does that still have to be the case?‘ Well, you can talk about it [severe pain] and I think most of them will hear you too […]”* (P9, 25 yrs., p. 125).

Study participants also stated that severe pain was a taboo subject, especially concerning the workplace, because, for example, people continued working despite the pain and did not take sick leaves.

*“And that maybe that’s also a taboo subject concerning professional life, especially if you have physical work. Because that’s how it’s done: Nothing is ever bad enough that you can’t go to work.“* (P4, 24 yrs., p. 53).

### Category eight: talking about it – ‘Contact with others in the same situation’

The final category summarised the need to encounter others and talk about needs and wishes.

By communicating about it, they could explain endometriosis, their experiences with it, and their perspective on it. Talking about endometriosis provided some relief and a way to express frustration and process personal experiences with the condition. The interviews indicated that there were participants who preferred to talk to people who care about endometriosis and pain. In addition, the women reported that, once they have been diagnosed with endometriosis, talking allowed them to explain the possible presence of the disease to others who also suffered from severe menstrual pain and had not (yet) been diagnosed.

The feeling of not being alone with the disease and the symptoms as well as talking to women who know what they were talking about and who understand them, was perceived as constructive and experienced as a positive experience. It was also possible for those affected to exchange experiences and tips.

*“It’s always good. Is always healing. Easy to hear you’re not the only one. That there are others who have problems.“* (P3, 33 yrs., p. 40).

*“In the past, I often had the feeling that it helps if you sit opposite a strengthened person […] Because when I was a teenager or something like that, as a younger adult, I would have needed someone to give me a bit of strength instead of sucking out the energy again and “bringing it over” together that things are so dirty.“* (P1, 31 yrs., p. 13).

## Discussion

In our study we conducted a comprehensive literature search to show, that no qualitative study on pain in women affected by endometriosis in Austria was done before. Prior, we published the first results that showed the experience and effects of pain, the methods for coping with these issues, and the needs of selected endometriosis people [40]. We interviewed ten participants and all of them stated a delay in diagnosis of up to ten years. This confirmed the results of another Austrian study [31].

A strength of this study is the qualitative approach. This allows us to explore the depths and multiplicity of women`s experiences in endometriosis. Our women reported negative thoughts about menstruation. Throughout the world, menstruation is seen as a private and intimate aspect of life that should be hidden from others which contributes to silence about problems with it and negative associations [41, 42]. Although the female cycle and monthly menstruation are not a taboo for affected women, some women have experienced that this topic is taboo in many places. Regarding the information given by women in our interviews and other studies, the conclusion can be drawn that, because endometriosis is associated with menstruation, the disease may be a taboo subject in society. Therefore talking about it with strangers, and non-affected people can be impossible [43]. A lack of understanding and knowledge about endometriosis are seen as factors that can increase silence and taboo about endometriosis [41].

Discussions with close people are often preferred since fertility and menstrual pain are sensitive topics. In addition, experiences are described by those affected that the constant addressing and expressing of severe pain is problematic, as it often leads to a lack of understanding by others. This leads to the interpretation that the reaction of others can be a reason for severe pain to become a taboo subject to talk about with individuals. Regarding future, it is crucial to make the disease endometriosis itself as well as menstruation, but also severe pain a topic within public and private discussions [43]. This can contribute to the removal of the taboo and assist in health-seeking behaviour for persons who suspect having the disease. Consequently, the diagnosis process could have started earlier and a shared decision-making process about effective treatment options could have started earlier too.

In our study women stated, to take on average six days per month off from work caused due to symptoms of endometriosis. That is confirmed by As-Sanie et al. (2019), who found out, that endometriosis patients are losing six hours per week at the workplace and five hours in productivity in personal households [44]. Bitzer et al. (2013) as well as Denny (2004) and Seear (2009) also showed that heavy menstrual flow has negative impacts on their attendance at work [11, 43, 45]. For affected women, it is difficult to talk about their health problems at the workplace as they do not feel to get understanding from employers.

For most of the women that were interviewed, it was helpful to talk to other affected women to understand the disease and not being alone with the symptoms. Prior studies have already suggested that those women should have contact with support groups in person and via social media [46–48].

Concerning their relationship with male partners in general, women reported a better understanding by partners after diagnosis. In our study, one woman broke up with her partner due to illness and stress. Similar results were reported in the systematic review of Young et al. (2015), which contained five studies that mentioned couples who broke up because of dyspareunia caused by endometriosis [14, 43, 49]. Data from a recent cross-sectional quantitative study in Austria and Germany [50] show the importance of male partner involvement in counselling women with endometriosis. They highlight an *“interdependence and reciprocal influence from both partners – positively and negatively –concerning psychological distress and sexual satisfaction […].”* (Schick et al. 2022:8). But, in addition, it needs to be mentioned, that in our collective, only heterosexual women participated.

Like cancer nurses, an endometriosis nurse seems to be an interesting speciality that could be developed to address women´s needs holistically. Resembling cancer schools, an endometriosis school could be developed in a participatory process with patients, caregivers and health professionals to create a new form of education and support.

A limitation of this study, that needs to be mentioned, is, that we only interviewed women with endometriosis. It would improve the research focus if women, who suffer from chronic pelvic pain were included. Hawkey et al. (2022) found out, that both groups had difficulties with fertility, work, and partnership as well as bad experiences with healthcare professionals. Furthermore, they even stated, that women with chronic pelvic pain have greater problems in communicating their complaints and fewer options in getting support from others [10]. Another important aspect that needs to be stressed is fertility and related problems – but that would have blown up the frame of this study as well as talking about religion and women affected by endometriosis in detail.

## Conclusion

To conclude, our data in the light of other studies show that the social environment plays a fundamental role in coping strategies in pain caused by endometriosis. Information about the disease, its course and treatment are important for family members and close friends, to create an understanding of endometriosis-related symptoms. Quality of life seems to improve slightly with measures within the social environment.

Comprehension of this chronic disease at the workplace also seems to be important. Involving occupational medicine and workplace health promotion departments in companies should be further goals to support affected women.

Women in our study and already published data reported that exchange with peers offers support. This opens a door for information events, patient organizations like support groups, and the inclusion of these in the supporting system.

Although a lot of time has passed since Seear (2009) claimed for more research on *“reasons for the persistence of stigma associated with menstruation and the impact that practices of menstrual etiquette that arise from such stigma have upon other health conditions experienced by women”*(Seear 2009:1226), we do not see a sufficient improvement in the last years and therefore these claims continue.

## Data Availability

The datasets used and/or analysed during the current study are available from the corresponding author upon reasonable request.
