# Correction: “I think to myself ‘why now?’” – a qualitative study about endometriosis and pain in Austria

**DOI:** 10.1186/s12905-023-02610-x

**Published:** 2023-08-28

**Authors:** Manuela Gstoettner, René Wenzl, Ines Radler, Margret Jaeger

**Affiliations:** 1 Department of Obstetrics and Gynecology, Medical University of Vienna, Waehringer Gürtel 18-20, Vienna, 1090 Austria; 2 Krankenhaus der Barmherzigen Schwestern, Seilerstätte 4, Linz, 4010 Austria; 3 Research Department of Education Centre of Social Fund Vienna, Schlachthausgasse 37, Vienna, 1030 Austria

Following publication of the original article [1], the surname and the family name for all the authors are corrected.

The original article has been corrected.
